# Detection and interval cancer rates during the transition from screen-film to digital mammography in population-based screening

**DOI:** 10.1186/s12885-018-4122-2

**Published:** 2018-03-05

**Authors:** Valérie D. V. Sankatsing, Jacques Fracheboud, Linda de Munck, Mireille J. M. Broeders, Nicolien T. van Ravesteyn, Eveline A. M. Heijnsdijk, André L. M. Verbeek, Johannes D. M. Otten, Ruud M. Pijnappel, Sabine Siesling, Harry J. de Koning, National Evaluation Team for Breast cancer screening, NETB

**Affiliations:** 1 Department of Public Health, Erasmus MC, PO Box 2040, Rotterdam, 3015 CN The Netherlands; 2 Department of Research, Netherlands Comprehensive Cancer Organisation (IKNL), PO Box 19079, Utrecht, 3501 DB The Netherlands; 3 Department for Health Evidence, Radboud University Medical Center, PO Box 9101, Nijmegen, 6500 HB The Netherlands; 4 Dutch Reference Center for Screening, PO Box 6873, Nijmegen, 6503 GJ The Netherlands; 5 Department of Radiology, University Medical Center Utrecht, Utrecht University, Utrecht, The Netherlands; 6 Department of Health Technology & Services Research, MIRA Institute for Biomedical Technology and Technical Medicine, University of Twente, PO Box 217, Enschede, 7500 AE, The Netherlands

**Keywords:** Breast cancer screening, Digital mammography, Screen-film mammography, Interval cancers, Detection rate, Programme sensitivity, Programme specificity

## Abstract

**Background:**

Between 2003 and 2010 digital mammography (DM) gradually replaced screen-film mammography (SFM) in the Dutch breast cancer screening programme (BCSP). Previous studies showed increases in detection rate (DR) after the transition to DM. However, national interval cancer rates (ICR) have not yet been reported.

**Methods:**

We assessed programme sensitivity and specificity during the transition period to DM, analysing nationwide data on screen-detected and interval cancers. Data of 7.3 million screens in women aged 49–74, between 2004 and 2011, were linked to the Netherlands Cancer Registry to obtain data on interval cancers. Age-adjusted DRs, ICRs and recall rates (RR) per 1000 screens and programme sensitivity and specificity were calculated by year, age and screening modality.

**Results:**

41,662 screen-detected and 16,160 interval cancers were analysed. The DR significantly increased from 5.13 (95% confidence interval (CI):5.00–5.30) in 2004 to 6.34 (95% CI:6.15–6.47) in 2011, for both in situ (2004:0.73;2011:1.24) and invasive cancers (2004:4.42;2011:5.07), whereas the ICR remained stable (2004: 2.16 (95% CI2.06–2.25);2011: 2.13 (95% CI:2.04–2.22)). The RR changed significantly from 14.0 to 21.4. Programme sensitivity significantly increased, mainly between ages 49–59, from 70.0% (95% CI:68.9–71.2) to 74.4% (95% CI:73.5–75.4) whereas specificity slightly declined (2004:99.1% (95% CI:99.09–99.13);2011:98.5% (95% CI:98.45–98.50)). The overall DR was significantly higher for DM than for SFM (6.24;5.36) as was programme sensitivity (73.6%;70.1%), the ICR was similar (2.19;2.20) and specificity was significantly lower for DM (98.5%;98.9%).

**Conclusions:**

During the transition from SFM to DM, there was a significant rise in DR and a stable ICR, leading to increased programme sensitivity. Although the recall rate increased, programme specificity remained high compared to other countries. These findings indicate that the performance of DM in a nationwide screening programme is not inferior to, and may be even better, than that of SFM.

**Electronic supplementary material:**

The online version of this article (10.1186/s12885-018-4122-2) contains supplementary material, which is available to authorized users.

## Background

Sensitivity and specificity are considered to be important quality assurance indicators for the performance of screening. The sensitivity of a breast cancer screening programme (BCSP) is calculated using the detection rate (DR) of screen-detected cancers and the interval cancer rate (ICR). The number of published studies that report interval cancers on a national level is scarce [1–4]. Data on nationwide interval cancers are difficult to obtain, as an accurate linkage between national screening data and the national cancer registry is required. In addition, because the number of interval cancers can only be determined at the end of an interval between screening rounds, there is an inherent delay in the availability of the data (usually two years), compared to data on cancers detected at screening.

In the past decade, many Western BCSPs made the transition from screen-film mammography (SFM) to digital mammography screening (DM) [5–9]. DM has been shown to influence the performance of BCSPs, leading to higher detection rates than SFM, through increased recall rates [6, 10–13]. In most studies, the increase in cancer detection was largely driven by a significant rise in the detection of DCIS. It has been argued that increased DCIS detection leads to a substantial rise in overdiagnosis of breast cancer without contributing to breast cancer mortality reduction. However, a recently published study showed an association between increased screen-detection of DCIS and fewer subsequent invasive interval cancer cases [14]. DM may thus also have the potential to lower ICRs.

In the Netherlands, the transition from SFM to DM was realised between 2003 and 2010 [15, 16]. In the same period, the percentage of 2-view mammography at subsequent screens increased from 50% to over 90% [17, 18]. Several Dutch studies showed statistically significant improvements in cancer detection for DM compared to SFM [13, 19–22], whereas others found no significant differences [16, 23]. However, so far, only regional interval cancer rates during the transition to DM in the Netherlands have been published [16, 21] and programme sensitivity on a national level was therefore not calculated.

The objective of this study was to evaluate the national performance of the BCSP in the Netherlands during the transition period to DM by assessing programme sensitivity and specificity, using screen-detected and interval cancers between 2004 and 2011.

## Methods

### Dutch breast cancer screening programme

The Dutch BCSP is carried out by 5 regional Cancer Screening Organisations (65 screening units), which invite all eligible women based on the population registry, aged 50–74 years, biennially to take part in screening. The attendance rate is around 80%. From 2003 onwards, a pilot phase started in which DM was introduced next to SFM, increasing the proportion of DM from 1% to 7% of all screens in 2007. This period was followed by a roll-out phase in which DM expanded from 10% in 2008, to 42% in 2009 and 100% in June 2010.

We collected data on all screens between 2004 and 2011. At initial screens 2-view mammography, with double reading, was performed. In 2004, about half of the subsequent screens had a second view and this proportion increased to 93% in 2010. The reading policy was double reading with consensus or arbitration. Women were only recalled if both independent readers concluded that the screening mammogram was positive or if a third reader came to this conclusion, in case of disagreement.

### Data

All screen-detected and interval cancers between 2004 and 2011 were analysed. To classify cancers as screen-detected or interval cancers, records of all screening examinations were linked to the Netherlands Cancer Registry (NCR). Linkages were made using an algorithm to identify identical subjects with high probability. The NCR classified the positive matches (94% of all breast cancers) preliminarily into screen-detected and interval cancers. Unclassified cancers were checked manually by the Cancer Screening Organisations, using information from the patient’s medical file. A small fraction of all women screened (0.01%) did not give permission to link their records.

Information on whether DM or SFM was performed was derived from the separate screening units, following the rollout schedule for digitization.

### Definitions

Screening examinations were defined as mammograms following an invitation to screening. These examinations were subdivided in initial screens, regular subsequent screens within 2.5 years after previous screening and irregular subsequent screens 2.5 years or later after previous screening (4% of all screens between 2004 and 2011). The latter were not used in this study: as the precise length of the irregular interval could not be determined from the aggregated dataset, including irregular subsequent screens would lead to distortion of (i.e. higher) detection- and interval cancer rates. Positive screens were considered to be screens with a suspicious mammographic lesion leading to recall and negative screens those without suspicious mammographic lesions, without any recommendation. Thus, screen-detected breast cancers were all diagnosed as a direct consequence of recall for further assessment, within one year after a positive screen.

All breast cancers diagnosed within two years after a negative screen were considered to be interval cancers. This concerned cancers arising from:

- Lesions that were screen-detectable at time of screening but were missed or not recalled
- Lesions that were present at screening but had minimal signs and were not recalled
- Lesions that were not present at screening and emerged within the screening interval

Interval cancers could also occur after a false-positive screen: if the cancer detected in the interval did not resemble the earlier detected lesion or was localized in the other breast, it was considered to be an interval cancer and coded accordingly. Interval cancers were thus calculated using all screens and not only women with a negative screen.

Both ductal carcinomas in situ (DCIS) and invasive cancers were included in the number of screen-detected and interval cancers.

We defined programme sensitivity as the number of screen-detected cancers expressed as a proportion of the total number of breast cancers diagnosed in women who were screened, within two years after screening (screen-detected cancers + interval cancers). Programme specificity was defined as the number of negative screens in women without breast cancer as a proportion of the total number of screens in women without a breast cancer diagnosis (true negatives + false-positives), within two years after screening. The false-positive rate was calculated as the number of recalls that did not lead to a breast cancer diagnosis per 1000 screens. As for some recalls the final diagnosis is not known, the numbers of true- and false-positives do not completely add up to the number of recalled women.

Age-adjusted recall (RR), false-positive (FPR), detection (DR) and interval cancer rates (ICR) per 1000 screens were calculated, using the total number of invitations during 2004–2011 as reference population. The positive predictive value (PPV) was calculated as the percentage screen-detected cancers (true positives) of all women recalled (true and false-positives). Performance indicators were based on all screening examinations (initial + regular subsequent), calculated by calendar year and age and presented with 95% confidence intervals (CI).

### Analysis

Screening examinations performed at age 75 (*N* = 9507) and interval cancers diagnosed within two years after screening at age 75 (*N* = 16) were excluded because of small numbers. Results are presented for the age group 49–74 and were calculated for the period 2004–2011, for all screening examinations and for DM and SFM screens separately.

Whether differences in outcomes were statistically significant was determined using the 95% confidence intervals. For proportions these intervals were calculated using the standard formula (P ± 1.96*s.e.). The 95% confidence intervals for the rates were calculated using a log linear model (exp(β+ log(N)); Poisson distribution) and rates were calculated per 1000 screens.

## Results

### All screens

#### Overall results

Between 2004 and 2011, 7,343,327 screens (initial + regular subsequent) were performed within the Dutch BCSP (Table 1). There were 41,662 breast cancers detected by screening; the DR was 5.7 per 1000 screens, of which 0.94 were DCIS. The recall rate (RR) was 17.8 per 1000 screens and the FPR 12.1 (PPV:33.5%). The 16,160 interval cancers identified led to an ICR of 2.2 per 1000, of which 0.1 were DCIS (Additional file 1: S3). The programme sensitivity was 71.4% and the programme specificity 98.8%.

**Table 1:** Age-adjusted results for all, DM and SFM examinations between 2004 and 2011 (49–74)

	All (95% C.I.)	DM (95% C.I.)	SFM (95% C.I.)
No. screens	7,343,327	2,620,442	4,722,885
No. screen-detected cancers	41,662	16,400	25,262
No. interval cancers	16,160	5748	10,412
No. false-positives	88,862	38,621	50,241
No. recalls	130,524	55,021	75,503
Recall rate	17.8 (17.7–17.9)	21.0 (20.8–21.2)	16.0 (15.9–16.1)
False positive rate	12.1 (12.0–12.2)	14.8 (14.7–15.28)	10.6 (10.5–10.7)
Detection rate (all)	5.7 (5.6–5.7)	6.2 (6.1–6.3)	5.4 (5.3–5.4)
Detection rate DCIS	0.94 (0.92–0.96)	1.1 (1.1–1.2)	0.83 (0.81–0.86)
Detection rate invasive	4.7 (4.7–4.8)	5.1 (5.0–5.2)	4.5 (4.5–4.6)
Interval cancer rate	2.2 (2.2–2.2)	2.2 (2.1–2.3)	2.2 (2.2–2.3)
Programme sensitivity (%)	71.4 (71.1–71.8)	73.6 (73.0–74.2)	70.1 (69.6–70.6)
Programme specificity (%)	98.8 (98.8–98.8)	98.5 (98.5–98.5)	98.9 (98.9–98.9)
Positive predictive value (%)	33.5 (33.2–33.7)	31.5 (31.1–31.9)	34.9 (34.5–35.2)

Rates are presented per 1000 screens

#### Trends over time

**Fig. 1:**
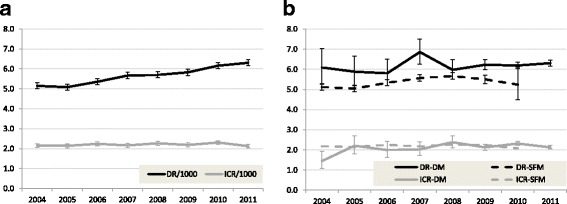
Age-adjusted detection and interval cancer rates per 1000 women screened for all screens (**a**) and DM or SFM^a^ (**b**) (49–74). ^a^In 2011 all screens were DM screens. Abbreviations: detection rate (DR); interval cancer rate (ICR); digital mammography (DM); screen-film mammography (SFM)

**Fig. 2:**
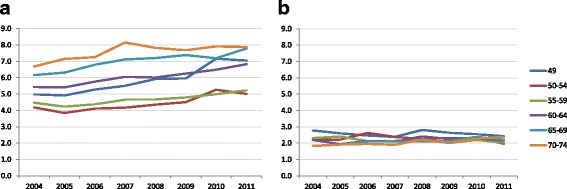
Age-specific detection (**a**) and interval cancer rates (**b**) per 1000 women screened

The DR significantly increased by more than 20% over the study period, from 5.1 per 1000 to 6.3 and the ICR remained stable (Fig. 1a; Additional file 1: S1a). The DRs of both DCIS (+ 0.5) and invasive breast cancers (+ 0.7) increased (Additional file 1: S1a). The detection rate increased for all age groups over the entire study period (Fig. 2a; Additional file 1: S2a). Detection also increased with age from 55 years onward; in the youngest ages (in particular 49 years) the detection rate was relatively high due to prevalent screening.

**Fig. 3:**
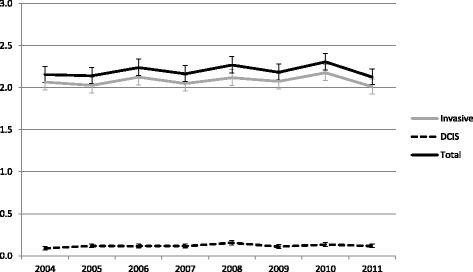
Age adjusted-interval cancer rate (per 1000 women screened) for all, invasive and in situ carcinomas

The overall ICR remained stable over the study period (2004: 2.2 per 1000 screens; 2011: 2.1; Fig. 1a; Additional file 1: S1a). The interval cancer rate showed a slightly decreasing tendency for the younger age groups over the study period and a slight increase in the trend for the older ages (Fig. 2b; Additional file 1: S2b). The fluctuation in the overall interval cancer rate was mainly determined by the rate for invasive breast cancers (Fig. 3). There were slight decreases in the age-adjusted overall interval cancer rate in 2007, 2009 and 2011 relative to the previous year (not statistically significant), accompanied by a decline in invasive interval cancers alone in 2007 and in both invasive and in situ interval cancers in 2009 and 2011 (Fig. 3; Additional file 1: S3).

**Fig. 4:**
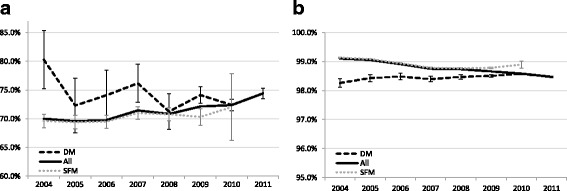
Aged-adjusted programme sensitivity (**a**) and programme specificity (**b**) for all screens, DM^a^ and SFM (49–74). ^a^The percentage DM screens between 2004 and 2007 was considerably small; in 2011, all screens were DM screens. N.B. scale Y-axis differs between graph a and b. Abbreviations: digital mammography (DM); screen-film mammography (SFM)

**Fig. 5:**
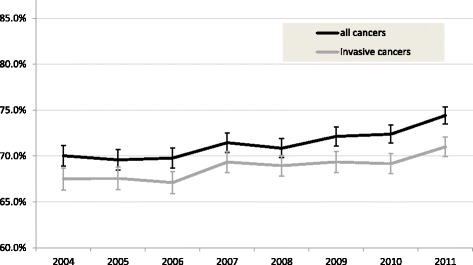
Age-adjusted programme sensitivity for all (invasive + DCIS) and invasive breast cancers only (49–74)

The programme sensitivity increased from 70.0% in 2004 to 74.4% in 2011 (Fig. 4a; Additional file 1: S1a) and increased statistically significant from 2010 (compared to 2004). The overall programme sensitivity was mainly determined by SFM between 2004 and 2008 and increased steeply with the expansion of DM between 2008 and 2011 (Fig. 4a; Additional file 1: S1b, S1c). The programme sensitivity strongly varied by age in 2004, which attenuated with the expansion of DM due to a significant increase in programme sensitivity for women aged 49–59 (Additional file 1: S4). Trends in programme sensitivity of all breast cancers and invasive cancers only were similar between 2004 and 2008 (Fig. 5). In 2009–2010, there was an increase in the sensitivity of all cancers but not of invasive cancers only, which reflects an increased detection of DCIS. In 2011, there was a similar rise in both groups, thus reflecting an increased detection of invasive cancers.

The RR increased significantly over time from 14.0 to 21.4 (Additional file 1: S1a). The programme specificity significantly declined slightly from 99.1% to 98.5% (Fig. 4b; Additional file 1: S1a). The difference in programme specificity between DM and SFM was most prominent in the beginning of the study period and decreased over time.

### DM versus SFM

#### Overall results

Of all screens, 2,620,442 were DM (36%) and 4,722,885 SFM (64%; Table 1). The RR for DM was 1.3 times higher than the RR for SFM. The DR was significantly higher for DM than for SFM (6.2 vs. 5.4), leading to higher programme sensitivity (73.6% vs. 70.1%). Both the DR of DCIS and invasive cancers were significantly higher for DM (1.1 and 5.1 respectively) than for SFM (0.83 and 4.5) (Table 1). The PPV and programme specificity were significantly lower for DM (31.5% and 98.5% respectively) than for SFM (34.9% and 98.9%). The ICRs were equal (2.2).

#### Trends over time

The DR of DM was higher than that of SFM in all years, and significantly higher in 2004, 2007 and 2009 (Fig. 1b; Additional file 1: S1b, S1c). The ICRs were similar over the years (except for 2004).

## Discussion

This nationwide study shows that the detection rate and programme sensitivity in the Dutch BCSP significantly increased during the transition from SFM to DM. This rise was most prominent for women under age 60. Despite the substantial improvement in detection, there was no decrease in the overall ICR. The programme specificity declined slightly as a result of the increased recall rate. Slight decreases were observable in the trend in interval cancers for younger women. The detection of both DCIS and invasive cancers and programme sensitivity were significantly higher for DM than for SFM, whereas the ICR was similar and the programme specificity was slightly lower for DM.

The increase in cancer detection can be partially explained by the transition to DM. Other studies also reported higher DRs for DM [6, 10, 12, 13]. DM has been demonstrated to lead to a substantially higher DCIS detection compared to SFM in the Netherlands [13, 20, 22]. There have been concerns that the increase in screen-detection of DCIS leads to overdiagnosis rather than to a significant additional reduction in breast cancer mortality [24]. Therefore, some might argue that the rise in breast cancer detection in this study largely reflects overdiagnosis. However, results of a recent study suggest that for every 1.5–3 screen-detected DCIS cases, one subsequent invasive interval cancer is averted; at levels of DCIS up to 1.5 per 1000 women screened (0.94 in our study) [14]. In addition, our findings show a significant increase in the detection of invasive breast cancers, which are less likely to be overdiagnosed than DCIS. Nevertheless, we recognize that a substantial rise in cancer detection may lead to a somewhat higher absolute number of overdiagnosed cases. Next to the transition to DM however, other factors also contributed to the increase in breast cancer detection. This increase already started in the mid-1990s, far before the introduction of DM [18]. First, the higher DR may also have resulted from an increase in the underlying breast cancer incidence over the years. It has been shown that the underlying breast cancer incidence in the Netherlands increased before the introduction of screening between 1975 and 1990 in women later invited to screening and in women not yet invited to screening (40–49) before and after the introduction of screening (1975–2004) [25], which has also been reported for other countries [26, 27]. It is reasonable to expect that the rise in background incidence continued after implementation of screening, due to increases in risk factors for breast cancer, including older age at first pregnancy and menarche and breast feeding at a later stage in life [28–30]. For example, in the Netherlands, the average age at birth of first child has increased from 26 years in 1970 to 29 years in 2004 [31].

Second, the significant increase in the percentage of 2-view mammography at subsequent screens during our study period (50% in 2005; > 90% in 2011 [18]) is likely to have contributed to higher breast cancer detection [17, 32, 33]. Finally, the DR may have increased due to changes in screening protocol. Following the outcomes of a study by Otten et al. [34], the national recall strategy was altered and the RR in the Netherlands increased from 0.9% in 2000 to 1.8% in 2007 [18].

We think that the stable interval cancer rate with the increasing trend in detection could also in fact reflect a reduction in the ICR, given the increase in background breast cancer incidence. The rise in detection may have prevented the interval cancer rate to increase as a result of increased breast cancer incidence.

Our estimate for the overall ICR (2.2 per 1000 screens) is in line with earlier reported rates from the BCSP in Germany (2.3) [35] and Norway (1.8) [2].

We found that DM performed significantly better than SFM in terms of DR and programme sensitivity, at the expense of significantly higher RRs and FPRs and slightly lower programme specificity. These findings are also consistent with results of earlier studies [6, 10, 12, 13, 19] . We found RRs (expressed as the percentage of screens recalled for further assessment) of 1.6% for SFM and 2.1% for DM throughout the study period. Recently reported RRs for DM in other European BCSPs range from 2.9% to 6.1% [5–7, 9, 36, 37]. Therefore, RRs in the Netherlands are still rather low compared to other countries [6, 12, 36, 38].

We did not find a difference in ICR between DM and SFM. Similar ICRs for DM and SFM were also reported for other BCSPs [37, 39]. It might be too early to observe the full effect of the transition to DM on the ICR. We observed a small, non-significant, decrease in the overall ICR in 2011 but we need future data, after a few years of full DM screening, to determine whether or not this will turn into a further statistically significant decline. Although we did not observe a significant difference in the overall ICR, looking at specific age groups we found that the ICR at initial screening in women aged 49–51 years was significantly lower for DM than for SFM (2.3 vs. 2.6 per 1000 screens; Additional file 1: S5). This finding corresponds to the results of the DMIST trial, which showed a higher diagnostic accuracy for DM than for SFM in pre- and perimenopausal women with dense breasts under the age of 50 [10].

The major strength of this study was the availability of national data on a large number of interval cancers. Thus, this study is the first nationwide analysis of sensitivity and specificity in the Dutch BCSP during the transition to DM. Furthermore, DM expanded during the second half of the study period and the effect of the transition from SFM to DM could therefore be studied well.

This study also had some limitations. Single screening examinations were not labelled as DM or SFM at time of screening and information about the proportion DM and SFM, during the years in which both modalities were used, had to be obtained from the screening units. The screens for which it was uncertain whether they were performed using screen-film or digital mammography were added to the screen-film group. This could lead to underestimation of detection rates for DM and to increased apparent detections rates for SFM. The difference in detection of DM relative to SFM could thus be (somewhat) greater than we report and our estimates may therefore be conservative. In addition, 2% of all breast cancers in the NCR database could not be classified as screen-detected or interval cancer.

## Conclusions

In conclusion, the detection rate in the Dutch breast cancer screening programme substantially increased between 2004 and 2011, whereas the interval cancer rate was stable over time. The recall rate increased over the study period, resulting in a decrease in programme specificity over time, even though the current specificity of the Dutch programme is still relatively high (in international context). DM resulted in higher detection rates than SFM, with similar interval cancer rates. The overall interval cancer rate, slightly, but non significantly declined in younger age groups and a significant rise in programme sensitivity in women under age 60 years was observed, which may be partly attributable to the transition to DM. Particularly young women may therefore have benefited from the change to DM but further exploration is needed to confirm these findings.

## Additional file

Additional file 1: Performance indicators of screening for women aged 49–74 and 49–51 years. Age-adjusted performance indicators per calendar year for all, screen-film and digital mammography screens and age-adjusted results for the age group 49–51 years. (PDF 107 kb)

## Abbreviations

BCSP: Breast cancer screening programme
DCIS: Ductal carcinoma in situ
DM: Digital mammography
DR: Detection rate
ICR: Interval cancer rate
PPV: Positive predictive value
RR: Recall rate
SFM: Screen-film mammography
