# Supplementary material for: Detection and interval cancer rates during the transition from screen-film to digital mammography in population-based screening
Source: BMC Cancer. 2018 Mar 5;18:256. doi: 10.1186/s12885-018-4122-2 (PMC5839006; doi:10.1186/s12885-018-4122-2)
Supplement: Supplementary file 1 — Performance indicators of screening for women aged 49–74 and 49–51 years. Age-adjusted performance indicators per calendar year for all, screen-film and digital mammography screens and age-adjusted results for the age group 49–51 years. (PDF 107 kb) [file 12885_2018_4122_MOESM1_ESM.pdf]

**S1a. Performance indicators (age-adjusted rates per 1000 screens) per calendar year for all screens (95% CI)**

| | Recall rate | Detection rate | Detection rate DCIS | Detection rate invasive | Interval cancer rate | Programme sensitivity | Programme specificity |
|---|---|---|---|---|---|---|---|
| 2004 | 14.0 (13.8-14.2) | 5.1 (5.0-5.3) | 0.73 (0.68-0.79) | 4.4 (4.3-4.5) | 2.2 (2.1-2.3) | 70.0% (68.9-71.2%) | 99.1% (99.1-99.1%) |
| 2005 | 14.5 (14.2-14.7) | 5.1 (4.9-5.2) | 0.74 (0.69-0.80) | 4.3 (4.2-4.5) | 2.1 (2.1-2.2) | 69.6% (68.5-71.0%) | 99.1% (99.0-99.1%) |
| 2006 | 16.1 (15.9-16.4) | 5.4 (5.2-5.5) | 0.88 (0.82-0.94) | 4.5 (4.3-4.6) | 2.2 (2.1-2.3) | 69.8% (68.7-70.9%) | 98.9% (98.9-98.9%) |
| 2007 | 18.0 (17.8-18.3) | 5.7 (5.5-5.8) | 0.84 (0.78-0.90) | 4.8 (4.7-5.0) | 2.2 (2.1-2.3) | 71.4% (70.4-72.5%) | 98.8% (98.7-98.8%) |
| 2008 | 18.2 (18.0-18.5) | 5.7 (5.6-5.9) | 0.87 (0.81-0.93) | 4.8 (4.7-5.0) | 2.3 (2.2-2.4) | 70.8% (69.8-71.9%) | 98.7% (98.7-98.8%) |
| 2009 | 19.1 (18.8-19.4) | 5.8 (5.7-6.0) | 1.02 (0.96-1.09) | 4.8 (4.7-5.0) | 2.2 (2.1-2.3) | 72.1% (71.1-73.2%) | 98.7% (98.6-98.7%) |
| 2010 | 20.2 (19.9-20.5) | 6.2 (6.0-6.3) | 1.2 (1.08-1.22) | 5.0 (4.9-5.2) | 2.3 (2.2-2.4) | 72.4% (71.4-73.4%) | 98.6% (98.6-98.6%) |
| 2011 | 21.4 (21.2-21.7) | 6.3 (6.2-6.5) | 1.2 (1.17-1.31) | 5.1 (4.9-5.2) | 2.1 (2.0-2.2) | 74.4% (73.5-75.4%) | 98.5% (98.5-98.5%) |

**S1b. Performance indicators (age-adjusted rates per 1000 screens) per calendar year for SFM (95% CI)**

| | Recall rate | Detection rate | Detection rate DCIS | Detection rate invasive | Interval cancer rate | Programme sensitivity | Programme specificity |
|---|---|---|---|---|---|---|---|
| 2004 | 13.7 (13.4-13.9) | 5.1 (5.0-5.3) | 0.73 (0.67-0.79) | 4.4 (4.3-4.5) | 2.2 (2.1-2.3) | 69.6% (68.5-70.8%) | 99.1% (99.1-99.2%) |
| 2005 | 14.1 (13.9-14.4) | 5.0 (4.9-5.2) | 0.75 (0.69-0.81) | 4.3 (4.2-4.4) | 2.1 (2.0-2.2) | 69.4% (68.2-70.6%) | 99.1% (99.1-99.1%) |
| 2006 | 15.8 (15.6-16.1) | 5.3 (5.2-5.5) | 0.89 (0.83-0.95) | 4.4 (4.3-4.6) | 2.3 (2.2-2.4) | 69.5% (68.4-70.6%) | 98.9% (98.9-99.0%) |
| 2007 | 17.6 (17.3-17.9) | 5.6 (5.4-5.7) | 0.83 (0.77-0.90) | 4.7 (4.6-4.9) | 2.2 (2.1-2.3) | 71.0% (69.9-72.1%) | 98.8% (98.8-98.8%) |
| 2008 | 17.9 (17.6-18.2) | 5.7 (5.5-5.8) | 0.88 (0.81-0.94) | 4.8 (4.6-4.9) | 2.3 (2.2-2.4) | 70.8% (69.7-71.9%) | 98.8% (98.8-98.8%) |
| 2009 | 17.5 (17.2-17.9) | 5.5 (5.3-5.7) | 1.00 (0.91-1.09) | 4.5 (4.3-4.7) | 2.2 (2.1-2.4) | 70.3% (68.9-71.8%) | 98.8% (98.8-98.8%) |
| 2010 | 16.2 (14.8-17.7) | 5.2 (4.5-6.1) | 0.74 (0.49-1.1) | 4.5 (3.8-5.3) | 2.1 (1.6-2.6) | 72.0% (66.2-77.8%) | 98.9% (98.8-99.0%) |
| 2011[a] | - | - | - | - | - | - | - |

[a]In 2011, all screens were digital. Abbreviations: screen-film mammography (SFM).

| S1c. Performance indicators (age-adjusted rates per 1000 screens) per calendar year for DM (95% CI) | | | | | | | |
|---|---|---|---|---|---|---|---|
| | **Recall rate** | **Detection rate** | **Detection rate DCIS** | **Detection rate invasive** | **Interval cancer rate** | **Programme sensitivity** | **Programme specificity** |
| 2004 | 23.3 (21.7-25.1) | 6.1 (5.3-7.0) | 0.87 (0.60-1.27) | 5.2 (4.5-6.1) | 1.4 (1.08-1.9) | 80.3% (75.2-85.4%) | 98.3% (98.1-98.4%) |
| 2005 | 21.4 (20.1-22.9) | 5.9 (5.2-6.7) | 0.64 (0.44-0.93) | 5.2 (4.6-6.0) | 2.2 (1.8-2.7) | 72.3% (67.6-77.0%) | 98.4% (98.3-98.6%) |
| 2006 | 20.8 (19.6-22.2) | 5.8 (5.2-6.5) | 0.73 (0.52-1.01) | 5.1 (4.5-5.7) | 2.0 (1.6-2.4) | 74.1% (69.7-78.5%) | 98.5% (98.4-98.6%) |
| 2007 | 22.7 (21.6-23.9) | 6.9 (6.3-7.5) | 0.85 (0.66-1.1) | 6.0 (5.5-6.6) | 2.0 (1.7-2.4) | 76.2% (72.8-79.5%) | 98.4% (98.3-98.5%) |
| 2008 | 21.1 (20.3-22.1) | 6.0 (5.5-6.5) | 0.79 (0.63-0.99) | 5.2 (4.8-5.7) | 2.4 (2.1-2.7) | 71.3% (68.2-74.3%) | 98.5% (98.4-98.6%) |
| 2009 | 21.0 (20.6-21.5) | 6.2 (6.0-6.5) | 1.1 (0.96-1.2) | 5.2 (5.0-5.4) | 2.1 (2.0-2.3) | 74.1% (72.7-75.6%) | 98.5% (98.5-98.5%) |
| 2010 | 20.3 (20.0-20.6) | 6.2 (6.0-6.4) | 1.2 (1.09-1.2) | 5.0 (4.9-5.2) | 2.3 (2.2-2.4) | 72.4% (71.4-73.4%) | 98.6% (98.6-98.6%) |
| 2011 | 21.4 (21.2-21.7) | 6.3 (6.2-6.5) | 1.24 (1.2-1.3) | 5.1 (4.9-5.2) | 2.1 (2.0-2.2) | 74.4% (73.5-75.4%) | 98.5% (98.5-98.5%) |

Abbreviations: digital mammography (DM).

**S2a. Age-specific detection rate per 1000 screens by calendar year**

| | 2004 | 2005 | 2006 | 2007 | 2008 | 2009 | 2010 | 2011 |
|---|---|---|---|---|---|---|---|---|
| 49 | 4.98 | 4.91 | 5.27 | 5.50 | 5.92 | 5.95 | 7.16 | 7.04 |
| 50-54 | 4.18 | 3.84 | 4.11 | 4.17 | 4.35 | 4.51 | 5.26 | 5.01 |
| 55-59 | 4.47 | 4.23 | 4.38 | 4.65 | 4.67 | 4.79 | 4.99 | 5.23 |
| 60-64 | 5.43 | 5.41 | 5.76 | 6.05 | 6.01 | 6.25 | 6.49 | 6.83 |
| 65-69 | 6.16 | 6.31 | 6.79 | 7.11 | 7.20 | 7.38 | 7.19 | 7.78 |
| 70-74 | 6.69 | 7.14 | 7.25 | 8.15 | 7.82 | 7.68 | 7.91 | 7.87 |
| **C.I. low** | | | | | | | | |
| 49 | 4.38 | 4.32 | 4.66 | 4.87 | 5.27 | 5.30 | 6.47 | 6.35 |
| 50-54 | 3.92 | 3.59 | 3.85 | 3.90 | 4.08 | 4.23 | 4.97 | 4.73 |
| 55-59 | 4.19 | 3.96 | 4.11 | 4.37 | 4.38 | 4.50 | 4.69 | 4.93 |
| 60-64 | 5.08 | 5.06 | 5.40 | 5.70 | 5.67 | 5.91 | 6.15 | 6.48 |
| 65-69 | 5.75 | 5.90 | 6.36 | 6.68 | 6.76 | 6.94 | 6.77 | 7.35 |
| 70-74 | 6.22 | 6.67 | 6.76 | 7.64 | 7.32 | 7.18 | 7.41 | 7.38 |
| **C.I. high** | | | | | | | | |
| 49 | 5.67 | 5.58 | 5.96 | 6.21 | 6.64 | 6.68 | 7.93 | 7.80 |
| 50-54 | 4.76 | 4.11 | 4.39 | 4.45 | 4.63 | 4.80 | 5.57 | 5.31 |
| 55-59 | 5.09 | 4.51 | 4.66 | 4.95 | 4.97 | 5.10 | 5.30 | 5.55 |
| 60-64 | 6.18 | 5.78 | 6.14 | 6.43 | 6.37 | 6.61 | 6.84 | 7.19 |
| 65-69 | 7.01 | 6.75 | 7.25 | 7.57 | 7.66 | 7.85 | 7.63 | 8.24 |
| 70-74 | 7.61 | 7.66 | 7.78 | 8.70 | 8.36 | 8.21 | 8.44 | 8.39 |

Abbreviations: confidence interval (C.I.)

**S2b. Age-specific interval cancer rate per 1000 screens by calendar year**

| | 2004 | 2005 | 2006 | 2007 | 2008 | 2009 | 2010 | 2011 |
|---|---|---|---|---|---|---|---|---|
| 49 | 2.77 | 2.60 | 2.47 | 2.38 | 2.80 | 2.64 | 2.54 | 2.42 |
| 50-54 | 2.21 | 2.21 | 2.63 | 2.38 | 2.25 | 2.31 | 2.33 | 1.96 |
| 55-59 | 2.31 | 2.41 | 2.12 | 2.14 | 2.19 | 2.01 | 2.19 | 2.03 |
| 60-64 | 2.20 | 1.93 | 2.14 | 2.09 | 2.41 | 2.25 | 2.30 | 2.15 |
| 65-69 | 1.83 | 1.92 | 2.09 | 2.10 | 2.09 | 2.12 | 2.39 | 2.20 |
| 70-74 | 1.82 | 1.91 | 1.95 | 1.90 | 2.22 | 2.02 | 2.27 | 2.38 |
| **C.I. low** | | | | | | | | |
| 49 | 2.33 | 2.18 | 2.06 | 1.98 | 2.37 | 2.22 | 2.14 | 2.04 |
| 50-54 | 2.02 | 2.02 | 2.42 | 2.18 | 2.06 | 2.11 | 2.14 | 1.79 |
| 55-59 | 2.12 | 2.21 | 1.94 | 1.95 | 1.99 | 1.83 | 2.00 | 1.84 |
| 60-64 | 1.98 | 1.73 | 1.92 | 1.89 | 2.20 | 2.05 | 2.10 | 1.96 |
| 65-69 | 1.61 | 1.70 | 1.86 | 1.87 | 1.86 | 1.89 | 2.15 | 1.98 |
| 70-74 | 1.59 | 1.67 | 1.71 | 1.66 | 1.96 | 1.78 | 2.02 | 2.12 |
| **C.I. high** | | | | | | | | |
| 49 | 3.30 | 3.10 | 2.96 | 2.87 | 3.32 | 3.14 | 3.02 | 2.89 |
| 50-54 | 2.63 | 2.42 | 2.85 | 2.59 | 2.46 | 2.52 | 2.54 | 2.15 |
| 55-59 | 2.75 | 2.62 | 2.32 | 2.35 | 2.40 | 2.22 | 2.40 | 2.23 |
| 60-64 | 2.61 | 2.16 | 2.38 | 2.31 | 2.65 | 2.47 | 2.52 | 2.35 |
| 65-69 | 2.18 | 2.17 | 2.36 | 2.36 | 2.34 | 2.38 | 2.65 | 2.45 |
| 70-74 | 2.17 | 2.18 | 2.23 | 2.17 | 2.52 | 2.31 | 2.57 | 2.67 |

Abbreviations: confidence interval (C.I.)

**S3.** Age standardised interval cancer rates for all cancers, invasive cancers and in situ cancers (95% confidence interval)

| | 2004 | 2005 | 2006 | 2007 | 2008 | 2009 | 2010 | 2011 |
|---|---|---|---|---|---|---|---|---|
| Invasive | 2.07 (1.97-2.16) | 2.03 (1.94-2.12) | 2.13 (2.03-2.22) | 2.05 (1.96-2.14) | 2.12 (2.03-2.21) | 2.07 (1.98-2.17) | 2.18 (2.09-2.27) | 2.01 (1.93-2.10) |
| DCIS | 0.09 (0.07-0.11) | 0.12 (0.10-0.14) | 0.11 (0.09-0.14) | 0.12 (0.10-0.14) | 0.15 (0.13-0.18) | 0.11 (0.09-0.13) | 0.14 (0.11-0.16) | 0.12 (0.10-0.14) |
| Overall | 2.16 (2.06-2.25) | 2.14 (2.05-2.24) | 2.24 (2.14-2.34) | 2.16 (2.07-2.26) | 2.27 (2.17-2.37) | 2.18 (2.09-2.28) | 2.31 (2.21-2.40) | 2.13 (2.04-2.22) |

**S4. Age-specific programme sensitivity of all screens (based on first two years after a screen)**

| | 2004 | 2005 | 2006 | 2007 | 2008 | 2009 | 2010 | 2011 |
|---|---|---|---|---|---|---|---|---|
| 49 | 64.2% | 65.4% | 68.1% | 69.8% | 67.8% | 69.3% | 73.8% | 74.4% |
| 50-54 | 65.4% | 63.4% | 61.0% | 63.6% | 65.9% | 66.1% | 69.3% | 71.8% |
| 55-59 | 65.9% | 63.7% | 67.3% | 68.5% | 68.1% | 70.4% | 69.4% | 72.1% |
| 60-64 | 71.2% | 73.7% | 72.9% | 74.4% | 71.3% | 73.6% | 73.8% | 76.1% |
| 65-69 | 77.1% | 76.7% | 76.4% | 77.2% | 77.5% | 77.7% | 75.1% | 78.0% |
| 70-74 | 78.6% | 78.9% | 78.8% | 81.1% | 77.9% | 79.1% | 77.7% | 76.8% |
| **C.I. low** | | | | | | | | |
| 49 | 59.3% | 60.5% | 63.4% | 65.1% | 63.4% | 64.9% | 69.9% | 70.5% |
| 50-54 | 62.9% | 60.8% | 58.5% | 61.1% | 63.4% | 63.7% | 67.1% | 69.6% |
| 55-59 | 63.5% | 61.3% | 64.9% | 66.1% | 65.7% | 68.0% | 67.1% | 69.9% |
| 60-64 | 68.6% | 71.2% | 70.5% | 72.1% | 69.1% | 71.4% | 71.8% | 74.2% |
| 65-69 | 74.5% | 74.2% | 74.0% | 74.9% | 75.2% | 75.4% | 72.8% | 75.9% |
| 70-74 | 76.0% | 76.4% | 76.3% | 78.8% | 75.4% | 76.7% | 75.3% | 74.4% |
| **C.I. high** | | | | | | | | |
| 49 | 69.2% | 70.3% | 72.8% | 74.4% | 72.3% | 73.7% | 77.7% | 78.2% |
| 50-54 | 67.9% | 66.0% | 63.5% | 66.1% | 68.3% | 68.5% | 71.5% | 74.0% |
| 55-59 | 68.3% | 66.2% | 69.8% | 70.9% | 70.5% | 72.8% | 71.8% | 74.3% |
| 60-64 | 73.8% | 76.2% | 75.4% | 76.6% | 73.5% | 75.7% | 75.8% | 78.0% |
| 65-69 | 79.6% | 79.2% | 78.9% | 79.5% | 79.8% | 79.9% | 77.3% | 80.0% |
| 70-74 | 81.2% | 81.4% | 81.3% | 83.4% | 80.3% | 81.6% | 80.0% | 79.1% |

Abbreviations: confidence interval (C.I.)

**S5. Age-adjusted results for all, DM and SFM between 2004 and 2011 for women aged 49-51**

| | **All** (95% C.I.) | **DM** (95% C.I.) | **SFM** (95% C.I.) |
|---|---|---|---|
| No. screens | 777908 | 273266 | 504642 |
| No. screen-detected cancers | 4667 | 1920 | 2747 |
| No. interval cancers | 1936 | 648 | 1288 |
| No. false-positives | 23682 | 10762 | 12920 |
| Recall rate | 33.6 (33.2-34.0) | 47.7 (46.9-48.6) | 31.9 (31.4-32.4) |
| False positive rate | 27.7 (27.3-28.1) | 40.4 (39.9-40.9) | 26.3 (25.9-26.8) |
| Detection rate (all) | 5.9 (5.7-6.1) | 7.3 (6.97-7.61) | 5.7 (5.5-5.9) |
| Detection rate DCIS | 1.1 (1.03-1.2) | 1.9 (1.81-2.00) | 1.2 (1.1-1.3) |
| Detection rate invasive | 4.8 (4.7-5.0) | 5.4 (5.2-5.6) | 4.5 (4.3-4.7) |
| Interval cancer rate | 2.5 (2.4-2.7) | 2.3 (2.2-2.4) | 2.6 (2.5-2.8) |
| Programme sensitivity (%) | 69.7 (68.6-70.8) | 76.3 (74.6-77.9) | 68.5 (67.1-70.0) |
| Programme specificity (%) | 97.2 (97.2-97.2) | 95.9 (95.8-96.0) | 97.4 (97.3-97.4) |
| Positive predictive value (%) | 16.5 (16.2-16.8) | 15.1 (14.9-15.4) | 17.5 (17.2-17.8) |

Rates are presented per 1000 screens
